# POLYMORPHISMS IN INFLAMMATORY CYTOKINES NOT ASSOCIATED WITH COGNITIVE TRAJECTORIES IN OLDER ADULTS

**DOI:** 10.1093/geroni/igad104.3529

**Published:** 2023-12-21

**Authors:** Karen Lawrence, Elana Gloger, Cristina Pinheiro, Frederick Schmitt, Suzanne Segerstrom

**Affiliations:** University of Kentucky, Lexington, Kentucky, United States; University of Kentucky, Lexington, Kentucky, United States; University of Kentucky, Lexington, Kentucky, United States; University of Kentucky, Lexington, Kentucky, United States; Oregon State University, Corvallis, Oregon, United States

## Abstract

Inflammation and key polymorphisms in inflammatory cytokine genes have been associated with increased Alzheimer’s disease (AD) risk. It is unclear if the same polymorphisms also predict domain-specific cognitive decline, that could eventually lead to AD/dementia, in otherwise healthy older adults. We investigated whether specific single nucleotide polymorphisms (SNPs) in three cytokine genes, IL-1β (rs16944; A or AA at -511), IL-6 (rs1800795; C or CC at -174), and TNFα (rs1800629; A or AA at -308), were associated with differential longitudinal trajectories of global cognitive function, episodic memory, attention and working memory, and executive function. Participants were 324 non-demented older adults (Mage=72.8 years at first visit, SD=6.09, age range 57-92 years) who completed 9 research visits, on average. Slopes were examined after adjustment for baseline age, sex, education, conversion to MCI or dementia during study, and APOE ɛ4 status. Findings indicated no statistically significant relationships between the SNPs of interest and longitudinal trajectories (spanning up to 16 years) of cognitive functioning in any cognitive domain examined. Other factors related to AD may be necessary for these SNPs to have significant brain-related effects. Attempts were made to further validate the non-significant findings by conducting post-hoc analyses to determine whether these SNPs were associated with plasma biomarker levels in this sample of healthy older adults. However, variability between and within the assays precluded confidence in the results. Further research is needed to determine whether these SNP genotypes are associated with concentrations of their respective blood-plasma biomarkers in non-demented older adults.

